# A Systematic Review of Community Engagement Outcomes Research in School‐Based Health Interventions

**DOI:** 10.1111/josh.12962

**Published:** 2020-11-12

**Authors:** Jaimie M. McMullen, Melissa George, Benjamin C. Ingman, Ann Pulling Kuhn, Dan J Graham, Russell L. Carson

**Affiliations:** ^1^ Associate Professor, (jaimie.mcmullen@unco.edu), University of Northern Colorado, Sport and Exercise Science, Gunter Hall 2640 Campus Box 39 Greeley Colorado 80639.; ^2^ Associate Director Prevention Research Center, Research Scientist, (melissa.george@colostate.edu), Colorado State University Human Development and Family Studies, Lake Street Offices 159 1508 Center Ave., Fort Collins, Colorado 80523.; ^3^ Director of Research & Evaluation, Research Assistant Professor (benjamin.ingman@du.edu), Center for Rural School Health & Education, Morgridge College of Education, University of Denver, 1999 E Evans Ave, Denver, Colorado 80210.; ^4^ Postdoctoral Research Fellow, (apullingkuhn@som.umaryland.edu), University of Maryland School of Medicine, Department of Pediatrics, 737 W. Lombard St, Room 169C Baltimore, Maryland 21201.; ^5^ Associate Professor, (dan.graham@colostate.edu), Colorado State University, 1876 Campus Delivery Fort Collins CO 80523.; ^6^ Research Advisor, (russ.carson@playcore.com), PlayCore, 544 Chestnut St., Chattanooga, Tennessee 37402.

**Keywords:** community involvement, community engagement, school health, Whole School, Whole Community, Whole Child model, school‐community partnerships

## Abstract

**BACKGROUND:**

Involving communities in school health has been purported as a practice integral to supporting a Whole School, Whole Community, Whole Child (WSCC) approach. Although community collaboration is often included in school‐based health initiatives, there is little research considering methods for increasing community engagement. The purpose of this study was to identify effective school‐based health interventions documenting changes in community engagement.

**METHODS:**

Academic experts and school stakeholders guided procedures for a systematic review of studies published from 1987–2017 and gray literature (ie, best practice documents; policy documents, etc.) on comprehensive school health interventions including community engagement as a targeted outcome.

**RESULTS:**

The search identified 9 studies addressing community as an outcome of school‐based health interventions; types of partnership mechanisms and partners' roles were classified.

**CONCLUSIONS:**

Although involving communities is a WSCC component and commonly recommended as a strategy fundamental to school health, there is little empirical research examining effective strategies for engaging communities and engagement is often not measured as part of intervention studies. Further measurement and research in engaging communities in school health is warranted.

School health promotion efforts, including the Whole School, Whole Community, Whole Child (WSCC) model specifically, have claimed that “community” is important when considering promotion of student learning, development, and health.^1^ The WSCC model refers to community within a specific component, called “community involvement”. In this work we use the term “community engagement” to honor the deeper levels of partnership and participation implied through the term engagement.^2^ Principles of community engagement have been developed and advanced by the fields of higher education,^3^ public health,^4^ social work,^5^ and primary/secondary education.^6^ Researchers have noted the benefits of community engagement for community partners,^7^ students of higher education,^8^ and K‐12 students and schools.^9^ Thus, the partnership between school and community is not unidirectional, rather it is viewed as a mutually beneficial partnership having implications for the broader context of the school and community. For example, schools not only benefit from having community organizations and agencies collaborating to provide resources for student and staff health and learning, but communities can benefit from having school staff, students, and their families contributing to the community through service‐learning, sharing school facilities and contributing to community members more broadly.^10^

Given the documented benefits of community engagement for both schools and communities, it is not surprising that community engagement has also been considered a catalyst for health promotion in school settings. The Coordinated School Health model and the WSCC model both position community involvement as a facilitator of student health and wellness. In the WSCC model, community involvement is included both as an individual component and also as a broader wrap‐around context guiding school practice. The WSCC Community Involvement component considers community groups, organizations, and businesses, and highlights both the role of the community in shaping the school, and the ways in which the school can participate in the community.^10^ State and national organizations also support the importance of involving community in school health promotion efforts.^11^, ^12^, ^13^

Despite the growing acknowledgement of community engagement's importance, and the continued advocacy for its inclusion across health‐related disciplines, there are still gaps in understanding community engagement in school settings. A systematic review of adolescent school‐based health interventions by Bush et al^14^ supports this tension, noting the “positive effect of involving parents and the community in health interventions”, but also stating that the results of these studies “were less clear‐cut about the *role* of community involvement” (p. 521). The need to address this uncertainty is particularly pressing given the role involving communities may play in fostering health equity.^15^ Overall, there exists a need to better understand both the role of community engagement in school‐based health interventions as well as community engagement as an outcome of such initiatives.

As Colorado implemented a large‐scale adoption of the WSCC model in 2016 driven by a collective impact approach supported by funding from a large foundation, a research collaborative, Advancing Innovation and Dissemination of Evidence‐Based Action in Schools (IDEAS) for school health, conducted systematic literature reviews for each of the 10 components of the WSCC model. The “Community Involvement” component systematic review identified school‐based health interventions that considered community involvement/engagement as an outcome measure. Through this manuscript, we recount the processes and outcomes of this systematic review, discuss patterns discerned from articles therein, and outline a research agenda for community engagement in school‐based health interventions.

## METHODS

### Participants

Interventions delivered to students in Kindergarten through 12th grade schools were included (ie, elementary and secondary schools), while studies of pre‐kindergarten schooling or post‐secondary schooling (colleges/universities) were excluded. Studies could include participants from low, middle, and high socioeconomic schools and schools in rural, urban, and suburban areas.

### Instrumentation

**Figure 1:**
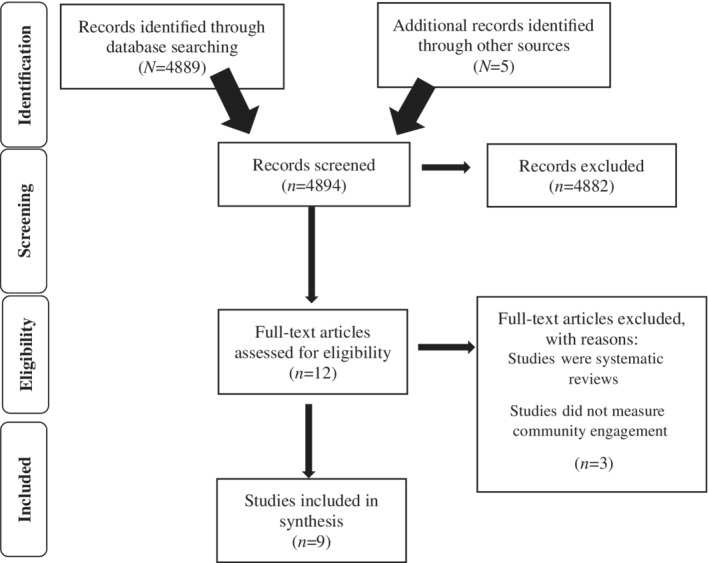
PRISMA Diagram^16^ Depicting the Different Phases of This Review

This systematic review utilized the Preferred Reporting Items for Systematic Reviews and Meta‐Analyses (PRISMA) 4‐phase flow diagram to display the processes of searching (Figure 1), inclusion, and exclusion.^16^ Search criteria permitted inclusion of interventions that used community engagement as the intervention and/or measured community engagement as an outcome. Search terms and databases were identified by Advancing IDEAS for School Health scientists who guided this work. The databases utilized were chosen due to their coverage of education, psychology, social sciences, health, and behavioral sciences domains and included the following databases: ERIC, Academic Search Premier, Sport Discus, Psychology & Behavioral Sciences, PsychInfo, Education Source, Social Science Citation Index, Cochrane Database of Systematic Reviews, and Education Proquest.^17^ Additionally, gray literature (ie, resources not published in academic journals; sometimes referred to as guidance or recommendation documents) and national experts in the field of community engagement in schools were consulted to identify articles. Studies were included in the review if they were effective peer‐reviewed school‐based community engagement interventions published between 1987 and 2017. It should be noted that one article was published in 2017 online head of print, but includes a print publication date of 2018. Qualitative studies and studies without a control group were considered. Only studies written in the English language were included.

### Procedure

The systematic search (Table 1) was conducted in 2017 and included: (1) school health search terms identified by IDEAS scientists, and (2) community engagement specific search terms identified by the aforementioned national experts in this content area. To determine study eligibility, the researchers reviewed titles, abstracts, and full texts of the selected papers as recommended by Booth et al.^17^

**Table 1:** Search Strategy

School Health Terms	Community Engagement Terms	
Comprehensive school health	+ Community Engagement, Community Involvement	+ in schools
Coordinated school health		
School Health		
School Wellness		
Healthy School*		

* Note. The phrase “in schools” was not included in searches with this term because of the presence of the term “school” in the phrase itself.

### Data Analysis

A narrative synthesis with tabular presentation was used to analyze and display the data. The narrative synthesis followed the guidelines from the Cochrane Consumers and Communications Review Group on data synthesis and analysis.^18^ The synthesis was conducted by considering components of the interventions that related to community engagement. Data regarding descriptive characteristics of the included studies were extracted as follows:

- Authors and year of publication.
- Community‐related objective, outcome, or aim (ie, community engagement was either identified as an objective, an outcome, an aim or a goal of the study/intervention)
- Description of community engagement (ie, information relating to the community's specific role within the intervention)
- Community partners (ie, details about community partners, including any entity or individual outside of the school building)
- Method of measuring community engagement (ie, how community engagement outcome was assessed)
- Community engagement findings or conclusions: (ie, relevant statements related to effects of including community engagement in the intervention)

Table 2 shows these results.

**Table 2:** Results Associated With School‐Based Intervention Studies Including a Community Engagement‐Related Outcome

Reference	Community‐Related Outcome/Aim/Goal	Partners	Description of Community Engagement	Measurement Community Engagement	Community Engagement Findings or Conclusions
Benjamins and Whitman^23^	Create and implement a culturally appropriate school‐based wellness program; form a school wellness committee (that includes community partners)	Local Jewish Federation, Sinai Urban Health Institute, the Associated Talmud Torahs, an Epidemiologist project director, a dietician, a mental health consultant, a social worker, and a joint school wellness council	Development, implementation and supervision of the intervention	Methods not specified	In order to change school curriculum, policies and practices, the community should be engaged in all aspects of intervention work—including needs assessment, project development, implementation and evaluation
Hoelscher et al.^24^	Effect of community involvement within an intervention	Unspecified “community partner”	Community partner was added to school‐based intervention committee to help with the development and support of school‐based initiatives	Process evaluation with a structured interview and self‐administered questionnaire	Community members were more involved in school‐based committees when a focus of the intervention; school‐based child obesity interventions that target disadvantaged schools are more effective when they include community partnerships to address and counteract disparities
Johnson‐Shelton et al.^25^	Develop a multi‐level research partnership through the community‐based participatory research (CBPR) model	School district, parents and families, 9 non‐governmental organizations (NGOs), multidisciplinary university researchers	Communities and Schools Together (CAST) project created a large partnership to facilitate school‐based health promotion for the prevention of childhood obesity	Process evaluation; methods not specified	Investigator‐initiated community partnerships can help to initiate and organize health‐promotion efforts
Ling et al.^27^	Promote family/community involvement	Healthy lifestyle coaches, county health departments, local hospitals, universities, and other community health‐related groups	Healthy lifestyle coaches implemented school‐based interventions; other partners were engaged through family fitness nights	Not specified	Schools should collaborate with community partners to conduct various school‐based health promotion events/activities, especially if they have budget limitations
Langhout et al.^19^	Assess the process and outcomes of a school‐community collaboration	Brighton Park Neighborhood Association, Woodson Friends, University members (ie, Partners for Progress)	Community involvement in a community garden project including development and maintenance of the garden which was based at a school	Process and outcome evaluation; methods not specified	A school‐based community garden can facilitate school‐community relations, but all entities need to be invested in order to achieve true collaboration
Tucker et al.^20^	Create a community partnership	University Nursing Students, Mayo Clinic, Public Health Services	Private and public health representatives participated in school‐based planning meetings prior to the intervention; nursing students implemented the intervention	Focus group interview	Establishment of a community partnership early in the project, and including multiple community partners can potentially improve the health of elementary school children
Waters et al.^21^	Develop sustainable positive changes in school, home and community environments (including community connections)	Government organization; community health services organization; university researchers	Partners were involved in the design, implementation and evaluation of a whole‐school program called *fun ‘n healthy in Moreland!*	School reported audit, principal exit interview, process evaluation using monitoring maps, photos and audits	In order to change policy, curriculum, behavior and health outcomes a long‐term partnership that includes community development is required
Wright et al.^26^	Evaluate the impact of a nurse coordinated, culturally and linguistically responsive comprehensive school health program	University representatives; parents; representatives from a local health clinic, city government, local library and from local business	Partners were involved through a community‐based participatory research approach in the development of the program and were included on a School Health Advisory Council	Not specified	Partnerships like ones developed through an Advisory Council can help with the integrity of school‐based programs especially as it relates to culture and development
Zahnd et al.^22^	Describe how an academic‐community partnership used community‐based participatory research principles to implement and maintain a recommended nutrition/physical activity curriculum	Springfield Urban League, Inc., school district (nurses and physical education coordinators), university school of medicine, state department of public health	Partners participated in collaborative meetings, coordinated the project and selected the curriculum	Not specified; described the partnership through a community‐based participatory research lens	An academic‐community partnership is effective when implementing and evaluating high school‐based obesity prevention programs

## RESULTS

Nine articles met the search criteria. Four articles^19^, ^20^, ^21^, ^22^ placed a community‐related outcome among their primary intervention goals; 4 others^23^, ^24^, ^25^, ^26^ identified such outcomes among their secondary aims. Further, while some studies measured the impact of engaging community in their intervention (eg, Hoelscher et al.^24^), others included a community component in their interventions, but did not evaluate the effects of this engagement (eg, Benjamins and Whitman^23^). It is important to note that none of the studies included in this systematic review had a singular focus of community engagement, rather each of the studies included multiple outcomes (most related to health and wellness of students in the school).

Community engagement was measured in a variety of ways across studies. Four studies included a process evaluation, but only 2 of these provided specific methods by which the process evaluation was completed.^21^, ^24^ Although 2 studies referred to interviews and/or self‐report measures,^20^, ^24^ only 1 study included other qualitative methods evaluating the impact of community engagement^21^ (ie, monitoring maps, photos, and audits).

There were also differences in how and where results were reported. While each of these studies included some measure of community engagement as an outcome, at times it was challenging to determine what the results were. Specifically, findings related to community engagement were often absent from papers' “results” sections, and were instead presented as statements in the conclusions—often without visible evidentiary support. For example, some such statements noted that schools should involve community when considering school‐based intervention work,^20^, ^22^, ^23^, ^24^, ^25^, ^26^, ^27^ that the inclusion of a community partner should be long‐term,^21^ and suggested that the partnership needs to be a true collaboration to succeed.^19^

Across the 9 studies, there were 6 different types of community partners engaging with schools. The most common partners were universities (N = 7),^19^, ^20^, ^21^, ^22^, ^25^, ^26^, ^27^ then government organizations including school districts (N = 6),^20^, ^21^, ^22^, ^25^, ^26^, ^27^ non‐governmental organizations (N = 4),^21^, ^23^, ^25^, ^27^ hospitals or medical centers (N = 4),^20^, ^22^, ^26^, ^27^ local organizations including neighborhood groups, businesses, libraries, etc. (N = 2),^19^, ^26^ and religious groups (N = 1).^23^ One study provided insufficient information to determine the type of community partner.^24^ The extent to which the level of community engagement was described also varied across studies; some dedicated substantial space to describing the community partner's role, and others provided little or no detail.

## DISCUSSION

### Characteristics of the Reviewed Community Engagement Literature

A relatively small number of published papers met the search criteria. While many school‐based health and wellness interventions include a community component,^14^ the present results highlight a gap in the literature: few studies consider community engagement not only as a strategy, but also as an outcome. This gap is particularly troubling when considering the plethora of recommendations for research and practice suggesting community engagement as an effective strategy to promote health and wellness in schools.^11^, ^12^, ^13^ In fact, several of the studies included in this review make claims regarding the effectiveness of community engagement in their own interventions often without a clear connection to study‐specific measurement or reported results. Further, the search criteria considered publications dating back to 1987, yet all appropriate articles were published in 2002 or later, with 8 of the 9 published between 2010 and 2018. The lack of studies on school‐based health interventions that explicitly include and measure community engagement among their intervention impacts is notable; this suggests a need to provide guidance and standard practices in several areas: (1) measuring community engagement, (2) determining/ reporting the extent to which community partners are active in the process, (3) measuring the feasibility, acceptability, and effectiveness of leveraging community partnerships for advancing school health and wellness initiatives.

Two patterns are evident when considering the results of this systematic review: one pattern concerns the nature of the partner, the second concerns the partnership's purpose. When considering the nature of the partner, the majority of the studies included a university partner. Two research teams engaged a university partner within a community‐based participatory research (CBPR) design;^22^, ^25^ both studies reported benefits of this type of model within the development and facilitation of school‐based interventions. Next most popular were government organizations, with 6 of 9 publications describing such partnerships. The specific government partners included district administrators, health and nutrition staff,^25^ county health departments,^20^, ^27^ government stakeholders,^21^ city government,^26^ and a state public health department.^22^

Whereas the clarity with which authors described the partners and their roles varied, many of the studies were deliberate in their selection of partners for a specific purpose related to the development, facilitation, and oversight of interventions (or all 3). For example, the partners described by Benjamins and Whitman^23^ were responsible for developing a culturally appropriate wellness program and joining an associated steering committee. Their partners, individuals from a variety of Jewish organizations, were strategic given the implementation of the wellness program within 2 Jewish schools. Lahghout et al.^19^ also describe the inclusion of community partners in order to facilitate culturally relevant project‐based learning. Zahnd et al^22^ ^(p. 466)^ referenced CBPR research principles as they discussed the “even level of value and respect for each partner” when considering partner knowledge, experience, and resource contributions. Through that CBPR lens, these authors provided a description of what each partner contributed, thereby clarifying each partner's role within the intervention. It is also suggested that an investigator‐initiated partnership can create “momentum, structure, and leadership in organizing multiple communities”^25^ ^(p 360)^ when considering health‐promoting initiatives in schools.

### Considerations for Research Related to Community Engagement in School Health Initiatives

Application of this systematic review protocol to the community engagement literature reveals a potential pathway for further inquiry as it pertains to health and wellness initiatives in school settings. Despite leading theoretical models recommending community engagement in school health as a best practice, there is little evidence that community engagement has been measured or its value assessed in more than 30 years of school health research literature. This gap leaves schools, communities, and researchers in a quandary about how to implement these recommendations for advancing school health initiatives through community engagement. As such, based on the scarcity of school‐based health intervention studies found in this systematic review, we identify 6 critical areas of need for advancing research to define, measure, and purposefully integrate community partners with school communities to obtain empirical evidence for community engagement in school health interventions. We suggest that the 6 areas of greatest need for advancing school health research are to: (1) develop shared understanding of “community” in community engagement, (2) develop shared understanding of community engagement, (3) develop conceptual models for engaging community, (4) develop metrics and assessment targets for community engagement, (5) apply lessons learned from closely related field, and (6) continue to solicit and incorporate community perspectives.

1. Develop a shared understanding of “community” in community engagement. Defining and describing community is necessary for developing a shared understanding of what is effective as a component of school‐based health intervention research. Therefore, there is a need to define who is the community within and outside of the school and in the broader community. There is little consensus on “who” community partners are, particularly around distinguishing family involvement from community involvement in schools, and school community versus non‐school community involvement. Based on the systematic review there exists a need for researchers to articulate who is considered part of the school versus part of the community in their interventions. There may be differences in the extent to which schools view district personnel or staff and professionals as “community” within or outside of their “school community” and understanding how schools define and/or separate school community versus the broader community may be important for identifying differences in structures, resources, and purpose for community engagement. Reviewing the existing studies highlighted uncertainty around defining community partners engaged in school‐based health interventions. For example, is it school‐based if the intervention utilizes community‐employed or community‐based staff? What if the community staff delivered interventions on campus, but after school? Developing definitions and language for studying and communicating about who constitutes a community partner is essential for growing our understanding of community engagement and measuring its impact on school health.

2. Develop shared understanding of community engagement. Defining and describing what comprises community engagement is key to developing a shared understanding of what engagement is effective and why as a component of school‐based health interventions. For example, outside of the semantic differences in terms like “community involvement” versus “community engagement” there exists an inherent lack of defining what school intentions are in building partnerships with community organizations and universities. This is evidenced not only by variation in description of how community partners were involved, if such a description was included in the publication at all, but also by our experience in communicating with community involvement/engagement researchers across the nation who informed the development of the systematic literature review process. In school health in particular, and in leading models advocating for community involvement (eg, WSCC), it is unclear what it means to involve, engage, and partner with agencies outside of the school building. The guidance provided is primarily to include community partners in school health efforts, but this lacks specificity (surely attributable to the lack of relevant research) on what this looks like, who is involved, in what capacity, for what duration, and in the context of what agreements or partnership arrangements. Defining and describing engagement is important as there is a continuum of collaboration or partnership that underlies involvement, and, according to the WSCC, a bidirectional relationship between school and community.

Advancing shared understanding of what community engagement means in school health is critically important for guiding research, practice, and policy efforts in supporting school health. Across the 9 studies in this review, many did not describe their conceptualization of community involvement/engagement. Future studies may contribute to our collective knowledge on community engagement by clearly describing how community is defined and the context of the particular school's perceptions of who constitutes the community. Researchers studying community engagement in school health must concretely articulate who was involved as a community member to begin advancing our ability to empirically test engagement's potential effectiveness in achieving school health and wellness benefits for students, staff, and community.

3. Develop conceptual models for engaging community. It is unclear from the existing research not only what is meant by community and engagement but also what role community engagement plays in advancing school health interventions. A lack of clarity in articulating the scope and role of community engagement in interventions complicates understanding the role that this involvement plays in facilitating beneficial outcomes. As evidenced in the results of this systematic review, community engagement can be framed as an element of the intervention, an outcome of the intervention, or both. Community involvement's role in school health is unclear. Clearly articulating the differences in how community partners are engaged and for what purpose is essential.

More explicit description of how and why individuals were involved in school health interventions will illustrate why certain individuals were classified as study participants, community partners, or collaborators. Attention to these concrete details is prerequisite to understanding the implications of involving certain partners on certain activities. Such approaches to delineating the purpose, processes, and potential benefits of community engagement in school‐based health interventions can provide empirical footing for conceptual models of community engagement. These models could then support and inform school‐based health initiatives and provide frameworks for the ongoing inquiry of community engagement in school health.

4. Developing metrics or assessment targets in community engagement. The continued development of a lexicon of community engagement would advance the field's discourse and practice. Shared community engagement constructs would provide a language and approach for both the practice and measurement of community engagement. Without such language, community engagement will continue to operate as an ambiguous dimension of school health initiatives, positioning it as a less intentional component of school health. Such an approach is in stark contrast to the explicit intentionality in shaping evidence‐based practices from other components of the WSCC model.

Work outside of school settings may provide insight in this regard given existing measurement and assessments of community engagement in other contexts. For instance, attempts to evaluate community engagement within organizations^28^ and community‐university partnerships^29^ may provide useful measurement and construct development opportunities to apply to school health. Particularly as it pertains to community involvement in the WSCC model, constructs of parental/family involvement,^30^ community partnerships,^31^ and community‐engaged development^32^ may also provide inroads to these lines of inquiry. Community engagement is often viewed as a multi‐dimensional construct in other fields (eg, education, psychology, and public health); thus, engagement may benefit from measurement development which reflects this multi‐dimensionality.

5. Apply lessons learned from closely related fields. The methods and strategies of related disciplines may be leveraged in the development of research on community engagement in school‐based health initiatives. While the possibilities of such a transdisciplinary exercise are innumerable, we thought it prudent to identify a few initial potential connections. Examples of evidence‐based processes for engaging community stakeholders in the fields of school mental health and prevention science may provide opportunities that school health can leverage or build examples from, as many of them include processes for team building, data‐driven decision making, and principles for partnership and stakeholder involvement. School interventions could benefit, for example, from considering the Communities that Care program. This intervention guides community coalition building through stages of assessment, capacity building, planning, implementing, and evaluating community and school programming based on community risk and protective factors.^33^ Additionally, leveraging infrastructure and processes which emphasize university‐community‐school partnership could lead to sustainable support for local community and school teams using evidence‐based programs in schools.^34^ Additionally, models of teaming in schools may be incorporated or offered as examples for involving community and schools through expanded school mental health models within school teams.^35^ The metrics used for measuring functioning of interdisciplinary teams could also be applied to the context of school‐community teams.^36^ Also, studies leaning on principles of community‐based participatory research will offer a variety of lessons about effective tactics and strategies of community involvement in school‐based partnerships. In fact, 2 of the works in this systematic review report following this approach^22^, ^25^

We may also borrow research methods used in related disciplines to better understand the role of community engagement in school‐based health interventions. For instance, dismantling trials, wherein select components are removed from multi‐component interventions,^37^ may illustrate the extent to which community involvement is responsible for beneficial outcomes.

6. Continue to solicit and incorporate community perspectives. We would be remiss not to identify the importance of including community perspectives and insight such that this research agenda complies with the same principles that guided the process of conducting this systematic review. The principles of community‐based participatory research^4^ and community engagement^38^ provide useful guideposts for pursuing this research agenda. Further, guiding principles suggested by the Patient Centered Outcomes Research Institute (PCORI) place stakeholders as equitable partners^39^—which is an important consideration for school‐based health intervention work.

There exists an innate paradox between offering an (inter)national research agenda on processes and procedures that should reflect and be shaped by the local communities pursuing these activities. As a result, a degree of eclecticism in the application and pursuit of this agenda is not only permissible, but encouraged. It is through the diverse and creative application of these ideals in numerous settings that the knowledge base on community engagement in school‐based health interventions will achieve new breadth and depth.

### Limitations

The purpose of this systematic review was to understand the existing evidence base for engaging communities in school‐based health interventions, and specifically considering the extent to which research involved communities and measured change in that community engagement across the school health intervention. Thus, this study included only those school‐based health interventions that attended to community engagement as an outcome and, as a result, does not include studies that included community engagement exclusively as a complementary dimension of school‐based health interventions. Moreover, the study criteria included studies that delivered interventions at school, which may have missed studies which more broadly conceptualized community engagement in school health as community partnering to deliver interventions to school communities. Lastly, only including studies published in the English language, it is possible that relevant international articles were overlooked.

### Conclusions

There exists ample general guidance for the use and promise of community engagement in school‐based health interventions. Community engagement is an identified practice component and undergirding context of the WSCC model. In this paper we reviewed the current state of the literature on community engagement in school‐based health interventions and offered recommendations for future inquiry on this topic.

## IMPLICATIONS FOR SCHOOL HEALTH

Engaging the community is integral to, and undergirds the WSCC approach, therefore understanding the effects of involving community in school‐based health initiatives is important. It is also imperative that we consider issues of equity and context given what we know about the connection between communities and overall health – therefore defining the role of the community, particularly in terms of equity, within school‐based health initiatives is underscored. Given the research and practice gaps that are revealed by this systematic review, it is challenging to provide specific recommendations for schools that are grounded in an abundance of evidence. However, the results of this systematic review do illustrate several themes or patterns that can be considered with respect to developing recommendations for schools. With that said, schools can use the information provided by this systematic review in the following ways:

- Seek out and/or enter into agreement for purposeful and mutually beneficial partnerships with universities, government agencies, non‐governmental organizations, neighborhood groups, religious groups, etc. to support school‐based health interventions.
- Consider your specific school context and issues of equity when seeking out or entering into a partnership agreement to support school‐based health interventions.
- While establishing a partnership, be sure to clearly develop roles for all partners involved in school‐based health interventions and revisit roles and expectations frequently.
- Be open‐minded when provided with suggestions for improving practice based on the results of school‐based health interventions.
- Engage actively, particularly in community‐based participatory research efforts.

Lastly, considering the recommendations for future inquiry and the suggestions for advancing research outlined in this systematic review will allow for more empirical evidence related to community engagement. As a result, increased engagement in this work has the potential to provide schools with information on how to engage with the community more successfully and vice versa.

### Human Subjects Approval Statement

This study did not involve human subjects.

### Conflict of Interest

All authors of this article declare they have no conflicts of interest.
